# Accurate Detection of *scutellata*‐Hybrids (Africanized Bees) Using a SNP‐Based Diagnostic Assay

**DOI:** 10.1002/ece3.70554

**Published:** 2024-11-17

**Authors:** Kathleen A. Dogantzis, Harshilkumar Patel, Stephen Rose, Ida M. Conflitti, Alivia Dey, Tanushree Tiwari, Nadine C. Chapman, Samir M. Kadri, Harland M. Patch, Elliud M. Muli, Abdulaziz S. Alqarni, Michael H. Allsopp, Amro Zayed

**Affiliations:** ^1^ Department of Biology York University Toronto Ontario Canada; ^2^ Behaviour, Ecology and Evolution Laboratory, School of Life and Environmental Sciences University of Sydney Sydney Australia; ^3^ Department of Animal Production and Preventive Veterinary Medicine, School of Veterinary Medicine and Animal Science São Paulo State University (UNESP) Botucatu São Paulo Brazil; ^4^ Department of Entomology The Pennsylvania State University State College Pennsylvania USA; ^5^ Department of Life Science South Eastern Kenya University (SEKU) Kitui Kenya; ^6^ Department of Plant Protection, College of Food and Agriculture Sciences King Saud University Riyadh Saudi Arabia; ^7^ Plant Protection & Health Agricultural Research Council Stellenbosch South Africa

**Keywords:** Africanized honey bee, *Apis mellifera*, biomonitoring, classification, machine learning

## Abstract

Hybrid populations of Africanized honey bees (*scutellata*‐hybrids), notable for their defensive behaviour, have spread rapidly throughout South and North America since their unintentional introduction. Although their migration has slowed, the large‐scale trade and movement of honey bee queens and colonies raise concern over the accidental importation of *scutellata*‐hybrids to previously unoccupied areas. Therefore, developing an accurate and robust assay to detect *scutellata*‐hybrids is an important first step toward mitigating risk. Here, we used an extensive population genomic dataset to assess the genomic composition of *Apis mellifera* native populations and patterns of genetic admixture in North and South American commercial honey bees. We used this dataset to develop a SNP assay, where 80 markers, combined with machine learning classification, can accurately differentiate between *scutellata*‐hybrids and non‐*scutellata*‐hybrid commercial colonies. The assay was validated on 1263 individuals from colonies located in Canada, the United States, Australia and Brazil. Notably, we demonstrate that using a reduced SNP set of as few as 10 loci can still provide accurate results.

## Graphical abstract

Africanized bees continue to pose a threat to the apiculture industry in North America. Our genetic assay when coupled with machine learning demonstrates high accuracy in correctly classifying target samples.

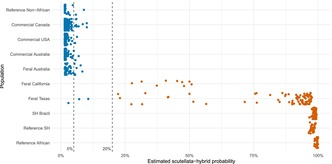

## 1 Introduction

The Western honey bee ( *Apis mellifera* ) has been translocated globally where it is extensively managed for its economic benefits, including wax and honey production, and more recently, its pollination services (Khalifa et al. 2021; Crane 1999). However, there have been widespread reports of declines in managed honey bee colonies (Potts et al. 2010; Pettis and Delaplane 2010; Smith et al. 2013; Gray et al. 2020), and in North America, there is additional concern about the displacement of colonies by the spread of hybrid Africanized bee (*scutellata*‐hybrids) populations (Huxel 1999; Lin et al. 2018). Consequently, significant efforts are underway to identify and address threats to prevent colony losses, including the development of accurate methods for identifying and tracking the movement of Africanized honey bees (*scutellata*‐hybrids).

*Apis mellifera* is native to Europe, Africa and parts of Asia, and can be delineated into at least seven genetically distinct groups comprised of the M‐lineage of Eurasia, the C‐lineage of Eastern Europe, the O‐ and Y‐lineages in Western Asia and the A‐, L‐ and U‐lineages of Africa (Dogantzis et al. 2021). The introduction of *A. mellifera* to North America is suspected to have occurred prior to the end of the 16th century, and to various regions in South America between the 18th and 20th centuries (Kent 1988; Carpenter and Harpur 2021; Crane 1999). These introductions began primarily with *A. m. mellifera* and *A. m. iberiensis* imported from Western Europe (M‐lineage) (Sheppard 1989; Kent 1988). Subsequent introductions were followed with *A. m. ligustica* and *A. carnica* from the C‐lineage, and *A. caucasica* from Western Asia (O‐lineage) (Kent 1988; Sheppard 1989). While apiculture quickly grew in North America, South American beekeepers found it difficult to establish hives and were dissatisfied with the low productivity of temperately adapted European ancestry bees (Kent 1988). Consequently, in 1956, the tropically adapted subspecies *A. scutellata* (A‐lineage) was introduced to Brazil with the intention of interbreeding them with previously introduced populations to establish a tropically adapted honey bee (Kerr 1967). Soon after the initial introduction, *A. scutellata* queens and drones were unintentionally released and hybridized with local populations producing feral “Africanized” bees (Winston 1992). We recognize the outdated term “Africanized” (Zarate et al. 2023), and hereafter refer to Africanized bees exclusively as *scutellata*‐hybrid honey bees.

Over nearly 70 years, *scutellata*‐hybrids have rapidly spread across South America to Northern Argentina (Porrini et al. 2019) and through Central America into the Southwestern United States (Rangel et al. 2016; Kono and Kohn 2015). Today, *scutellata*‐hybrid honey bees are the most abundant managed and feral honey bee population across this region. The rapid and successful expansion of these honey bees has been attributed to a combination of ecological and behavioural factors that contribute to higher fitness in *scutellata*‐hybrids relative to European‐hybrid populations (Schneider, Degrandi‐Hoffman, and Smith 2004; Winston 1992). For example, *scutellata*‐hybrids have retained many of the behavioural and physiological traits prominent among African (A‐lineage) subspecies, including faster colony growth and a greater tendency to abscond and swarm (Schneider, Degrandi‐Hoffman, and Smith 2004, Winston 1992). Aggressive colony defence, a notorious trait among *scutellata*‐hybrid bees, is also enhanced through hybridization with existing European populations (Harpur et al. 2020; Zayed and Whitfield 2008). These traits, while advantageous in tropical habitats, can make these populations less favourable for beekeeping in other regions.

During their expansion in the Americas, *scutellata*‐hybrid honey bees are thought to have displaced or hybridized with previously abundant European colonies, radically changing the genetic composition of populations (Pinto et al. 2005; Rangel et al. 2016; Whitfield et al. 2006). Established *scutellata*‐hybrid populations are comprised largely of African ancestry, which represents on average 75% of individual genetic composition (Chapman et al. 2015; Nelson et al. 2017; Zayed and Whitfield 2008; Zárate et al. 2022). The remaining ancestral proportions originate primarily from the M‐lineage, with some contribution from the C‐lineage (Chapman et al. 2015; Nelson et al. 2017; Zárate et al. 2022). However, ancestral proportions are variable across the population distribution. This is especially prevalent at the current northern and southern range extent, where the proportion of African ancestry exhibits a gradient between 5% and 77% (Calfee et al. 2020; Zárate et al. 2022).

The rapid and dynamic spread of *scutellata*‐hybrids populations illustrates the potential risk of their invasion to regions currently free of *scutellata* genetics. Although there is evidence to suggest that *scutellata*‐hybrids may have reached their range limit at temperate latitudes (Calfee et al. 2020; Porrini et al. 2019), changes in the environment could improve habitat suitability, thus promoting the movement of the invasive population (Jarnevich et al. 2014; Stohlgren et al. 2014; Gill and Sangermano 2016). For example, recent studies have shown that *scutellata*‐hybrids are slowly expanding their distribution into regions that serve as queen breeding hubs for North America (Cridland et al. 2018; Lin et al. 2018). As such, to prevent the incorporation of undesirable phenotypes into other commercial colonies, several countries, including Canada and Australia, have implemented import restrictions from regions with known *scutellata*‐hybrids. However, without the ability to accurately detect and track the movement of *scutellata*‐hybrids, there remains a risk of accidentally importing populations, especially from recently colonized regions.

Traditional methods of identifying *scutellata*‐hybrid samples can be inaccurate and have the potential to misidentify samples. Errors are often a result of the variability in ancestry proportion, which can confound conclusions based on morphology (Guzmán‐Novoa, Page, and Fondrk 1994) and maternally inherited mitochondrial DNA sequences (Sheppard and Smith 2000). However, current diagnostic tools using single nucleotide polymorphisms (SNPs) have demonstrated excellence in differentiating honey bee subspecies (e.g., Henriques, Browne, et al. 2018, Henriques, Parejo, et al. 2018; Parejo et al. 2016; Muñoz et al. 2017, 2015; Pinto et al. 2014). To identify *scutellata‐hybrids*, diagnostic assays have been developed using 95 and 37 SNP loci, which estimate the proportion of African lineage ancestry among samples (Chapman et al. 2017, 2015). While these SNP assays are a significant improvement over traditional methods, they have some drawbacks: (1) The current 95 and 37 SNP diagnostic assays were developed using only three out of the seven ancestral lineages, and consequently, do not benefit from recent large‐scale genomic datasets on *A. mellifera* subspecies (Dogantzis et al. 2021). (2) SNPs for these assays were not chosen based on an information criterion, and studies have shown that markers selected by information content outperform randomly selected SNPs (Muñoz et al. 2017). (3) The reliance on ancestry proportion thresholds for detecting *scutellata*‐hybrids can be confounded by the variance in ancestry among samples (Calfee et al. 2020; Zárate et al. 2022).

Novel approaches, such as the use of machine learning, could greatly increase the efficacy of diagnostic assays. The use of machine learning in population genomic analyses is an emerging trend and has already demonstrated success in identifying selective sweeps, inferring demographic histories (Schrider and Kern 2018), and has been used to discern between several *A. mellifera* subspecies (Momeni et al. 2021). Notably, supervised machine learning algorithms, which utilize prior knowledge to make predictions about new data points, are ideal for classification tasks. Here, we present an improved SNP diagnostic assay and a classification model designed to identify *scutellata*‐hybrids. The first aim of this study involved categorizing native honey bee samples into their respective ancestral lineages and determining the genetic composition of managed honey bees. This initiative helped determine ancestry informative markers that may be effective in discriminating lineages. Next, we constructed a random forest classifier to subsample perspective loci and rank markers based on their informativeness for effectively classifying *scutellata*‐hybrids. A diagnostic assay was constructed based on 113 informative loci and was validated using 1263 honey bee samples collected from North America, South America and Australia. Classification of samples based on genotyping results was estimated with a support vector classifier, which estimates the classification probability of a sample to a predetermined group. Overall, the diagnostic assay provides an accurate means for identifying individual honey bee samples and has the potential to provide accurate results with a reduced set of informative markers.

## 2 Methods

### 2.1 Genome Sequence Processing and SNP Detection

Our dataset consists of 243 previously sequenced honey bee genomes from the species' native range (Haddad et al. 2015; Harpur et al. 2014; Fuller et al. 2015; Wallberg et al. 2017; Dogantzis et al. 2021), 16 newly sequenced *scutellata*‐hybrids and six North American honey bees, one of which was previously published (Mcafee et al. 2016); total dataset (*N* = 265) (Table S1). Sample preparation and genome sequencing of unpublished *scutellata*‐hybrids (Dogantzis et al. 2021) and North American samples (Harpur et al. 2014) followed previously published protocols. Sequence reads were trimmed of Illumina adapters and low‐quality bases (< 20) using Trimmomatic v0.36 (Bolger, Lohse, and Usadel 2014), and were retained for downstream assembly if > 50 and > 35 bps in length from 100 to 150 and 50 bp Illumina sequencing data respectively. Reads were aligned to the *A. mellifera* reference genome (Elsik et al. 2014) using NextGenMap aligner v0.4.12 (Sedlazeck, Rescheneder, and Von Haeseler 2013). BAM files were sorted using SAMtools v1.3.1 (Li et al. 2009) and reads were marked for duplicates using Picard v2.1.0 (https://broadinstitute.github.io/picard/). Base quality scores were recalibrated using GATK v3.7 BaseRecalibrator (Van Der Auwera et al. 2013) using previously identified variants as reference (Harpur et al. 2014, 2019). SNPs were identified with GATK v3.7 (Poplin et al. 2017; Van Der Auwera et al. 2013) using HaplotypeCaller and filtered using VariantRecalibrator using previously identified variants as reference (Harpur et al. 2014, 2019). Additionally, we used the following hard filter thresholds: MQ < 40.0, QD < 5.0, FS > 11.0, MQRankSum −2.0 < *x* > 2.0 and ReadPosRankSum −2.0 < *x* > 2.0 (Dogantzis et al. 2021). Variants were excluded if they failed two or more of the filters. In addition, we excluded variants located within five base pairs of an indel or areas of low complexity (Harpur et al. 2019), and excluded variants from the unmapped scaffolds.

### 2.2 Population Structure of Reference Samples

ADMIXTURE v1.3.0 (Alexander and Lange 2011) was used to estimate ancestry proportions and population structure of the 265 honey bee genomes. This analysis was performed with 1 M randomly selected bi‐allelic markers with a minor allele frequency of > 0.10 among at least one of the predicted subspecies (see Dogantzis et al. (2021)). ADMIXTURE was run with predicted *K* values 1–18 using the 10× cross‐validation procedure. Additionally, a principal component analysis (PCA) was generated to examine the genetic clustering of samples. The PCA was constructed with the SNPRelate (Zheng et al. 2012) package in R v3.6.0 (R Core Team 2021) using SNP markers with a minor allele frequency > 0.10 among at least one of the predicted subspecies.

### 2.3 SNP Selection and Assay Design

To choose a comprehensive set of diagnostic markers for the SNP assay, we employed a two‐step selection process. First, we calculated pairwise measures of F_ST_ (Weir and Cockerham 1984) between genetically distinct honey bee lineages using VCFtools v0.1.17 (Danecek et al. 2011). SNPs of interest were bi‐allelic, had a variant call rate greater than 95% and were highly differentiated (*F*_*ST*_ > 0.8) between the African (A)‐lineage and the remaining lineages, as *scutellata*‐hybrids predominantly (> 75%) consist of A‐lineage ancestry.

Second, to further reduce the dataset, we used a random forest classification model (Breiman 2001) to determine the informativeness of SNP markers in *scutellata*‐hybrid classification (Figure S1). Any missing genotypes were imputed using the consensus genotype from the lineage of origin. On average, 0.12 ± 0.51 loci on the final SNP assay have been imputed among the training samples. Final genotypes were coded as “0” representing homozygous reference, “1” representing heterozygous and “2” representing homozygous alternative. To train the random forest classifier, we divided the 265 honey bee genomes into a training group and a testing group, which contained 177 (66%) and 88 (33%) samples respectively (Table S1). We ensured the testing and training groups had an approximately equal proportion of samples from each lineage and the commercial populations. We used the GridSearchCV option as implemented in scikit‐learn Python package (Pedregosa et al. 2011) to determine the optimal parameters of the random forest classifier, including n_estimators, max_features and max_depth. The model was run for 30 replicates and the feature importance for each replicate was estimated for the top markers using the feature_importances_ option as implemented by the scikit‐learn package (Pedregosa et al. 2011). Overall, there were 824 markers of interest that were present two or more times across replicates or were ranked among the top 75th percentile of the scored features.

We developed a three‐panel Agena iPLEX Gold SNP array estimated to hold approximately 120 SNP markers (Figure S1). To increase the design success of the panel, markers were submitted for inclusion on the array if they were free of secondary SNPs at least 16 bp up or downstream of the target loci and were greater than 5000 bp apart from other informative loci to reduce linkage disequilibrium. Of the 824 top‐ranking SNPs, 249 markers fit the preceding requirements and 113 markers were successfully designed for the panel (Table S3). Panel design, production and validation, in addition to oligo design, were completed at the Genome Quebec Innovation Centre (Quebec, Canada). Prior to final development, the testing samples were used as an independent validation of the predictive accuracy of the selected markers.

### 2.4 Sample Processing and SNP Genotyping

To validate the diagnostic assay, 1263 samples were collected from North America, South America and Australia (Table S2). The dataset included honey bees collected from known managed commercial colonies in Canada (*N* = 841) (Harpur et al. 2015), and honey bees from known managed commercial (*N* = 88) and unmanaged feral (*N* = 49) colonies from Australia (Chapman et al. 2016). Additional samples were collected from known managed commercial colonies from the United States (*N* = 115), including three *Varroa*‐resistant strains (Chapman et al. 2015). Samples representing *scutellata*‐hybrids included honey bees from managed colonies in Brazil (*N* = 78) that were established as wild‐caught swarms (Harpur et al. 2020; Kadri et al. 2016). The validation dataset also included honey bees from known unmanaged feral colonies from Texas (*N* = 83) (Chapman et al. 2015) and honey bees from nine colonies in California, suspected to be *scutellata*‐hybrids, but whose colony origin is unknown (Table S2). Commercial samples from Canada, Australia and the United States, and feral honey bees from Australia were known non‐*scutellata*‐hybrids. Feral honey bees from Texas and California were presumed *scutellata*‐hybrids, while honey bees from Brazil were known *scutellata*‐hybrids. Hypothesized classifications are outlined in Table S2. This dataset was supplemented with an additional 29 reference *scutellata*‐hybrid honey bee genomes whose corresponding genotypes were extracted from published variant data (Kadri et al. 2016). These samples were labelled as reference *scutellata*‐hybrid and were included as part of the known reference testing group (Table S2).

DNA extraction was performed on honey bee samples using Mag‐Bxind Blood & Tissue DNA HDQ 96 Kit (Omega Bio‐Tek Inc. USA) optimized for KingFisher Flex Purification System (Thermo Fisher Scientific Inc. USA). For tissue lysis, either half or whole bee thoraces were flash frozen in liquid nitrogen and finely ground using a pestle. We then added 350 μL Tissue Lysis Buffer and 20 μL Proteinase K, and heated samples overnight at 55°C. After processing with the KingFisher System, samples were eluted in nuclease‐free water (Thermo Fisher Scientific Inc. USA) to a final volume ranging from 50 to 80 μL. DNA was quantified using NanoDrop 2000 Spectrophotometer (Thermo Fisher Scientific Inc. USA). DNA quality was assessed with 1.0% agarose gel electrophoresis. SNP genotyping was outsourced to Genome Quebec Innovation Centre (Quebec, Canada).

### 2.5 Assay Validation

To test the functionality of the assay, all validation samples were genotyped at 113 loci and were analyzed using a linear support vector classifer (SVC) (Figure S1). The model was trained using the training dataset (*n* = 177) (Table S1), and then was tested on the validation samples (*N* = 1263) and the previously genome‐sequenced honey bees of known origin (*n* = 117) (*n* = 88 testing samples and *n* = 29 reference *scutellata*‐hybrids) (Table S2). To run the model, genotypes were coded as “0” representing homozygous reference genotypes, “1” representing heterozygous genotypes and “2” representing homozygous alternative genotypes. The SVC was trained using a linear kernel with the GridSearchCV option to estimate optimal parameters for C as implemented by the scikit‐learn package (Pedregosa et al. 2011). The model was fit using the following parameters: kernel = “linear,” *C* = 0.001, class_weight = balanced and probability = true. Classification probabilities of unknown samples to a *scutellata*‐hybrid origin were computed with the predict_proba option using the scikit‐learn package (Pedregosa et al. 2011). Missing genotypes were imputed conservatively as “2”, representing the genotype associated with the African (A)‐lineage as this model does not easily accommodate missing values. On average, 1.38 ± 2.46 loci were imputed among the validation samples.

### 2.6 Diagnostic SNP Reduction and Imputation Simulations

To test the model performance with a reduced set of diagnostic loci, we retrained the linear SVC on random subsets of 10–70 loci over five replicates. Each model was trained using the training dataset (*n* = 177) and tested on 117 known reference samples (*n* = 88 testing samples and *n* = 29 reference *scutellata*‐hybrids) and 694 validation samples that were originally successfully genotyped across all 80 loci (*n* = 54 commercial Australia, *n* = 559 commercial Canada, *n* = 67 commercial USA, *n* = 11 feral Australia and *n* = 3 *scutellata*‐hybrids Brazil) (Table S2). For simplicity, samples originally predicted to have a probability assignment > 90% to *scutellata*‐hybrid classification were labelled as *scutellata*‐hybrids (*n* = 91 *scutellata*‐hybrids and *n* = 720 non‐*scutellata*‐hybrids).

To evaluate the effects of imputation on probability estimates, we randomly imputed 1 through 80 of the informative markers with the African (A)‐lineage genotype (“2”) and regenerated probability estimates for each iteration. The analysis focused on predictions of non‐*scutellata*‐hybrids to determine the false‐positive rate associated with imputation. The linear SVC was trained using the training dataset (*N* = 177) and tested on 720 samples (*n* = 29 reference non‐African, *n* = 54 commercial Australia, *n* = 11 feral Australia, *n* = 559 commercial Canada and *n* = 67 commercial USA) (Table S2). For simplicity, all 720 samples were labelled as non‐*scutellata*‐hybrids.

### 2.7 Comparison Between SVC and ADMIXTURE

To test the accuracy of the support vector classifier (SVC), we compared our results to those generated using traditional ancestry proportion methods. Here, we generated a supervised ADMIXTURE v1.3.0 (Alexander and Lange 2011) model using 80 SNPs and with *K* = 2 clusters representing *scutellata*‐hybrid or non‐*scutellata*‐hybrid. The model was supervised using the training dataset (*n* = 177) (Table S1) and then ancestry proportions (*Q* values) were estimated on the validation samples (*N* = 1263) and the previously genome‐sequenced honey bees of known origin (*n* = 117) (Table S2). Although we are not estimating true ancestry proportions, the *Q* values were used as proportional assignments to a *scutellata*‐hybrid or non‐*scutellata*‐hybrid classification. As such, we compared the probability estimates generated from the SVC (see above for Section 2) to the Q proportions generated using ADMIXTURE.

## 3 Results

### 3.1 Ancestry Estimation of Reference Samples

**FIGURE 1:**
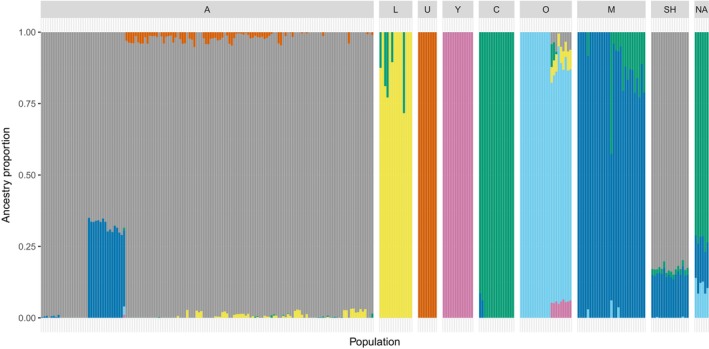
Patterns of admixture and ancestry among honey bee lineages and populations. Patterns of ancestry and admixture for *N* = 243 native *A. mellifera* samples grouped into their respective lineages (Africa (A, L and U), Western Asia (Y and O), Eastern Europe (C), Eurasia (M)), *n* = 16 hybrid *scutellata*‐hybrid honey bee samples (SH) and *n* = 6 commercial North American honey be samples (NA). Vertical bars represent individual bees and coloured segments represent the proportion of ancestry estimated to *K* = 7 genetic clusters. *Scutellata*‐hybrid honey bees exhibit an average A‐lineage ancestry of 82.7%, M‐lineage ancestry of 15.3% and C‐lineage ancestry of 2%. In contrast, North American honey bees consist of an average of 73.1% C‐lineage ancestry, with an average of 12.9% M‐lineage ancestry and 11.0% O‐lineage ancestry.

Our dataset comprises 243 previously sequenced honey bee genomes from the native range of *A. mellifera* . Genetic clustering results produced by the program ADMIXTURE, illustrate that when *K* = 7, native honey bee samples clustered into previously identified lineages, including *A. m. unicolor* (U‐lineage) from Madagascar and *A. m. lamarckii* (L‐lineage) from Egypt (Dogantzis et al. 2021) (Figure 1). When the structure analysis is conducted with *K* predictive values 3–6 (Figure S2), we observe gradual separation of clusters with increasing *K* between the C‐ and O‐lineages and the A‐, L‐, U‐ and Y‐lineages. These patterns are also reflected in the PCA analysis which depicts the proximate clustering of the A‐, L‐, U‐ and Y‐lineages together, while the C‐ and O‐lineage cluster closer together (Figure S3). While most samples have a definitive lineage assignment (> 80% to a single ancestry), the structure results highlight that several individual honey bees have low‐to‐moderate levels of admixture, likely a result of hybridization with geographically neighbouring lineages.

Additionally, we used the native honey bee samples as reference lineages to estimate the ancestral proportions of reference North American (*N* = 6) and *scutellata*‐hybrid honey bees (*N* = 16) (Figure 1). Individuals from the reference *scutellata*‐hybrid population had a large portion of their ancestry originating from the A‐lineage, representing on average 82.7%. The remaining genetic composition was comprised of M‐lineage ancestry and C‐lineage ancestry contributing an average of 15.3% and 2% respectively. The PCA results also emphasize the extensive introgression of A‐lineage ancestry into *scutellata*‐hybrids, depicted by the proximate clustering of these groups (Figure S3). North American samples can also be classified as admixed, with an average of 73.1% of ancestry originating from the C‐lineage, and M‐ and O‐lineages contributing an average of 12.9% and 11.0% respectively. Similarly, the North American population clusters most closely with the C‐lineage samples in the PCA analysis, reflective of shared ancestral origins (Figure S3).

Based on the results of the clustering analyses, it was observed that the honey bee lineages in Africa (A, L and U) and the Y‐lineage in West Asia exhibit a higher degree of genetic similarity, supported by a recent common ancestor (Dogantzis et al. 2021). Thus, given these lineages comprise or cluster proximately with *scutellata*‐hybrids, these samples were used to represent potential *scutellata*‐hybrid genetic diversity. Similarly, samples from North America are genetically composed of the M‐, C‐ and O‐lineages, thus these samples were used as representatives for commercial honey bee diversity (non*‐scutellata*‐hybrids). These classifications were maintained for SNP selection and panel validation (see Section 2 for details).

### 3.2 Assay Validation and Sample Classification

We developed an SNP‐diagnostic assay designed to identify *scutellata*‐hybrid honey bees. SNP selection occurred via a two‐step process using F_ST_ to identify highly differentiated SNPs and a random forest classifier to determine marker informativeness (see Section 2 for details). The final diagnostic assay consists of 113 informative markers, of which 87 could be successfully genotyped across 1263 validation samples collected from South America, North America and Australia. We removed an additional five loci due to a low call rate (< 80%), and two markers due to monomorphic genotype calls, resulting in 80 informative SNPs (Table S3). Using the genotypes determined by the SNP assay, we used a linear SVC (support vector classifier) to estimate the probability of a *scutellata*‐hybrid classification. The model was trained on 177 training samples and was then tested on 1263 validation samples and 117 reference samples whose genomes were previously sequenced (Table S2). Metrics of model performance can be found in the Supporting Information Material (Figure S4).

Based on estimations from the model, the honey bee samples from Canada were classified as *scutellata*‐hybrids with an average probability of 1.87% (range 1.5%–10.1%) (Table 1, Table S2). This indicates a < 2% chance of being a *scutellata*‐hybrid, or > 98% chance of not being a *scutellata*‐hybrid. The exception was one sample that had a probability of 38.4% (not shown); likely due to 10 loci that failed genotyping and were conservatively imputed with an African lineage genotype. When the SVC model is retrained and tested without the missing loci, the classification of this sample to a *scutellata*‐hybrid origin is 18.1%. This sample was subsequently removed from the proceeding analyses due to low genotyping coverage. The honey bee samples from Australia were classified as *scutellata*‐hybrids with probabilities ranging from 1.5% to 8.3% (Table 1), and honey bee samples from Brazil were classified as *scutellata*‐hybrids with probabilities between 96.6% and 99.4%. We detected a wide variance in the classification of feral honey bee populations collected in North America. Among feral populations in Texas, the average probability of a *scutellata*‐hybrid classification was 82.9% but ranged from 2.9% to 97.7%, while feral populations in California averaged 58% and ranged from 49.2% to 72.4% (Table 1).

**TABLE 1:** Probability estimates to a *scutellata*‐hybrid (SH) classification. The table provides the upper probability estimate, the lower probability estimate and the mean probability estimate for each population.

Population	*N*	Lower probability	Upper probability	Mean
Commercial Canada	840	1.49%	10.09%	1.87%
Reference non‐African	29	1.51%	8.95%	2.31%
Commercial USA	115	1.51%	6.26%	1.97%
Commercial Australia	88	1.51%	6.91%	2.05%
Feral Australia	49	1.51%	8.35%	2.86%
Feral Texas	83	2.94%	97.65%	82.86%
Feral California	9	27.63%	50.84%	42.03%
Reference SH	33	96.37%	99.08%	97.83%
SH Brazil	78	96.60%	99.44%	98.64%
Reference African	55	98.56%	100.00%	99.55%

**FIGURE 2:**
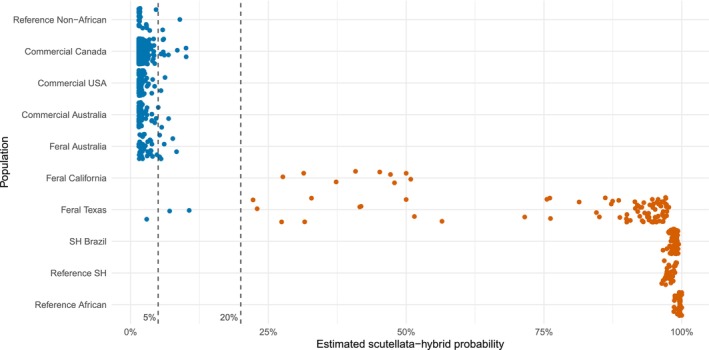
Classification of validation samples using a probability threshold. When a 20% estimated *scutellata*‐hybrid probability threshold is applied, all samples above this value are considered *scutellata*‐hybrids. A 20% threshold produced a 0% false‐negative and false‐positive rate among the following sample groups: reference samples, commercial samples, feral Australia and *scutellata*‐hybrid (SH) Brazil. Among samples labelled Feral California and Texas, 97% of samples are classified as *scutellata*‐hybrid. The dashed lines indicate a 5% and 20% threshold, blue dots represent samples with < 20% probability of a *scutellata*‐hybrid classification, while orange dots present samples with > 20% probability of a *scutellata*‐hybrid classification.

Given the extensive range of probabilities estimated by the model, it is essential to determine an acceptable threshold by which samples are designated *scutellata*‐hybrids. As such, we determined the functionality of the diagnostic assay by measuring the false‐negative and false‐positive rates at various probability thresholds for known samples. If a strict threshold of 5% is used, there is a 2% false‐positive rate among samples labelled reference non‐African, commercial and feral Australia, but a 0% false‐negative rate among samples labelled reference African, reference *scutellata*‐hybrid (SH) and *scutellata*‐hybrid (SH) Brazil. When a 20% threshold is used, there are no false positives or false negatives (Figure 2). Among samples labelled feral, a 20% probability threshold results in 97% of samples classified as a *scutellata*‐hybrid (Figure 2).

### 3.3 Reduction in Diagnostic Loci

**FIGURE 3:**
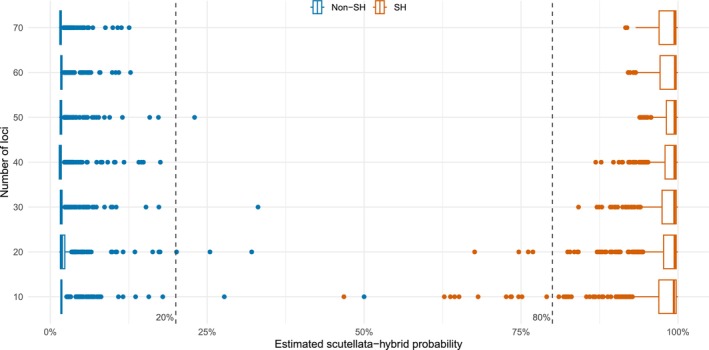
Effects of SNP reduction on probability estimates. Classification probabilities were estimated for 811 samples (*n* = 91 *scutellata*‐hybrids, *n* = 720 non‐*scutellata*‐hybrids) using random subsets of 10–70 loci, replicated five times. Results show a boxplot of the probabilities predicted for non‐*scutellata*‐hybrids (Non‐SH) and *scutellata*‐hybrids (SH) at each iteration. Dotted lines represent 20% and 80% probability estimates.

Although the diagnostic panel genotypes at 80 informative markers, there may be circumstances where the number of loci available for classification is reduced. To evaluate how a reduction in diagnostic markers impacts classification, the model was retrained on random subsets of 10–70 loci and then tested on 811 samples (*n* = 91 *scutellata*‐hybrids, *n* = 720 non‐*scutellata*‐hybrids). This process was replicated five times to capture variability across loci. Results showed that when ≥ 30 diagnostic markers were used, all *scutellata*‐hybrids were estimated with a probability above 80%, while all but two non‐*scutellata*‐hybrids were estimated with a probability below 20% (Figure 3). Employing a 20% classification threshold results in a false‐negative rate of 0 across all replicates (*n* = 2275) and a false‐positive rate of 0.01% across all replicates (*n* = 18,000). In the most extreme scenario when only 10 loci are used, 13 *scutellata*‐hybrids were estimated with probabilities below 80% but maintained a 0% false‐positive rate at a threshold of 20%. Among non‐*scutellata*‐hybrids, there were two samples assigned probability estimates above the 20% threshold (0.06% false‐positive rate, *n* = 3600) (Figure 3).

### 3.4 Effect of Imputation on Classification

**FIGURE 4:**
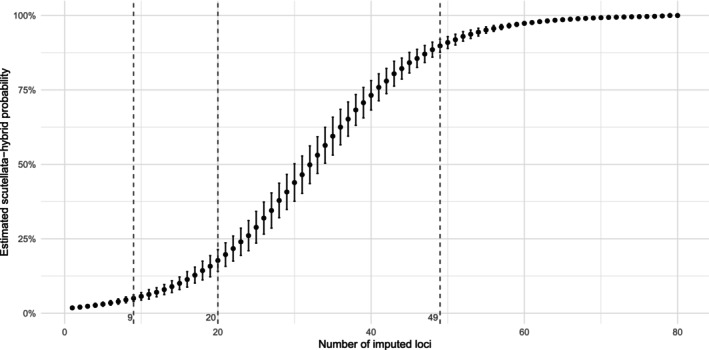
Effects of imputation on probability estimates. To evaluate the effects of imputation on classification, 1–80 loci were randomly imputed as the African (A‐lineage) genotype among 720 non‐*scutellata*‐hybrid samples. The curve depicts the average probability estimate to a *scutellata*‐hybrid classification for each iteration of imputed loci. Bars represent the standard deviation of estimates, and dotted lines represent iterations where 9, 20 and 49 loci were imputed.

The linear SVC model used to classify samples does not easily accommodate missing values, as such, missing genotypes are coded conservatively as the representative genotype for African (A‐lineage) ancestry. To evaluate the impact of imputation on probability estimates, we randomly assigned genotypes representative of African ancestry among 1–80 loci across 720 non‐*scutellata*‐hybrid samples and then determined the false‐positive rate for each iteration. We found that when ≤ 9 loci were imputed, all samples were classified as *scutellata*‐hybrids with probabilities below 20%. This resulted in a false‐positive rate of 0% when using a classification threshold of 20% (Figure 4). When 10–17 loci are imputed, we find that on average, three samples fall above the 20% threshold (0.28% false‐positive rate), with probabilities ranging between 20% and 36% (average 25.5%). There is a considerable increase in false‐positive rate (23.3%) when ≥ 20 markers are imputed, and when ≥ 49 markers are imputed, all samples are classified as *scutellata*‐hybrids with probabilities above 80% (Figure 4).

### 3.5 Comparison Between SVC and Proportion Methods

The traditional approach to categorizing *scutellata*‐hybrids relies on estimating ancestry proportions using software such as ADMIXTURE (Alexander, Novembre, and Lange 2009) and Structure (Pritchard, Stephens, and Donnelly 2000). Although these models are informative and effective, the SNP assay developed here results in a binary classification that is not directly informed by ancestry proportions of the samples. Despite this, we conducted a supervised ADMIXTURE analysis using the training samples and estimated ancestry proportions (*Q* values) for the testing and validation samples. We compared the ancestry proportions (*Q* values) to the probabilities estimated from the SVC model to identify differences in classification. The average estimate of a *scutellata*‐hybrid classification for non‐*scutellata*‐hybrid samples was comparable between models, with individual samples differing by an average of 2% (Figure S5A). However, the variance in estimates was higher for ADMIXTURE, and using a 20% threshold would result in false positives among reference non‐African and commercial honey bees from Canada (Figure S5A). Marked differences in classification were identified among the validation and reference *scutellata*‐hybrids from Brazil and the feral bees from Texas, where individual samples differed by an average of 13.4% between models (Figure S5B). Overall, classification estimates from the SVC model were higher for *scutellata*‐hybrids and exhibited lower variance compared to estimates using ADMIXTURE (Figure S5B), showing a stronger inclination toward *scutellata*‐hybrid identification.

## 4 Discussion

As *scutellata*‐hybrid honey bees continue to expand their range, diagnostic tools that minimize classification errors are needed to improve the detection and monitoring of these hybrid populations. Here, we developed a SNP diagnostic assay that, when coupled with a support vector classifier (SVC), can consistently and effectively identify *scutellata*‐hybrids with high probabilities (> 80%) and minimal false negatives (0%). The SVC model works by finding the hyperplane that maximizes the separation between classes—in this case, *scutellata*‐hybrid or non‐*scutellata*‐hybrid. Classification probabilities are estimated using Platt scaling (Platt 1999) on the SVC scores, which represent the distance of a sample to the hyperplane. These probabilities range from 0 to 1 and indicate the likelihood of the classification. When testing samples of known origin, the model can classify samples into the correct group with a probability estimate greater than 80%. Of the 1263 validation samples classified, only 1.9% of samples did not meet an 80% probability estimate for a single group. These samples primarily belong to unmanaged colonies, such as the feral bees from Texas and California, which showed a wide variance in probability estimates (2.9%–97.7%) to a *scutellata*‐hybrid classification. The broad range of estimates suggests that this cohort contained *scutellata*‐hybrid honey bees, commercial honey bees of European origin and several samples that had intermediate levels of introgression. Although the model did not definitively classify all samples to a single group (< 2% overall or 25% of feral bees), we can still categorize these bees as *scutellata*‐hybrids if they fall above the proposed 20% threshold. Since our methodology integrates assisted machine learning models, additional reference samples of confirmed *scutellata*‐hybrid origins, including those with variable levels of hybridization, can be added to improve classification. At present, we are limited to the training set produced in this study, which does not currently reflect the full range of genetic diversity found across honey bees and *scutellata*‐hybrids.

Genotyping issues can complicate classification tasks by reducing the available data. To mitigate instances of missing data, we recommend removing loci when a considerable number of samples are affected. For example, a call rate threshold of 80%–97% is commonly applied (Henriques, Browne, et al. 2018; Howe et al. 2020); in this study, all retained loci had a call rate of > 88%. Despite this potential loss of data, we demonstrated that reduced subsets of SNP markers retained the ability to classify samples with high predictive probabilities. Notably, when as few as 30 markers are used, all *scutellata*‐hybrid and all but two non‐*scutellata*‐hybrid samples are correctly classified with a 20% probability threshold. This results in a 0% false‐negative and 0.01% false‐positive rate. The development of reduced SNP panels (Muñoz et al. 2015; Henriques, Browne, et al. 2018, Henriques, Parejo, et al. 2018; Chapman et al. 2017) is a common goal as they can provide cost savings and reduce the computational demand typically associated with large SNP panels or genome sequencing. Alternatively, if only a few samples are affected by missing genotypes, it is recommended to either omit the sample or conservatively impute genotypes representative of African (A)‐lineage origins. Imputation on fewer than 20 markers does not adversely affect classification and retains probability estimates above 80%. Additionally, the results from the imputation analysis revealed a conservative bias toward positively identifying *scutellata*‐hybrid honey bees. For instance, when ≥ 49 loci are imputed as the African (A)‐lineage genotype, samples are assigned a *scutellata*‐hybrid classification with ≥ 80% probability. This conservative bias presents a twofold advantage by decreasing the chance of false negatives and being sensitive toward moderate levels of introgression.

The accuracy of the diagnostic assay can be attributed to several factors considered during the design process. For instance, SNPs were evaluated on their discriminant power based on measures of F_ST_ and feature importance was estimated with a random forest classifier. Previous studies have shown that diagnostic assays constructed with SNPs chosen using an information criterion perform better than those chosen at random (Muñoz et al. 2017), and loci with high measures of F_ST_ are substantially advantageous for population discernment (Henriques, Parejo, et al. 2018; Chapman et al. 2015; Willing, Dreyer, and Van Oosterhout 2012; Muñoz et al. 2015). Markers that are highly differentiated are less likely to be lost to genetic drift and are more likely to reach fixation in a population. The random forest classifier added an extra measure of scrutiny to markers by measuring SNP informativeness, emphasizing markers that best differentiate *scutellata*‐hybrids from non‐*scutellata*‐hybrids. The designed binary outcome of the assay removes the reliance on ancestry proportions and allows for discrete classification. Although a variety of models could be deployed for classification, supervised models tend to be more accurate. Here, a support vector classifier was chosen as this model shows high accuracy, precision and fast classification speed (Osisanwo et al. 2017) and can undergo periodic retuning and retraining with new data to ensure it adapts to changing patterns. In this study, the SVC model effectively performed binary classification, provided informative probability estimates and performed well with a reduction in SNPs. The model also outperformed traditional ancestry proportion methods by estimating higher classification probabilities, especially for suspected and known *scutellata*‐hybrids, and provided zero false‐positive and false‐negative rates using a 20% threshold.

In conclusion, we show that 80 SNP markers when combined with machine learning can clearly and effectively classify samples by providing a non‐ambiguous probability estimate for a *scutellata*‐hybrid identification. This is advantageous over previous methods that rely on morphology or ancestry proportions, which can introduce uncertainly, especially among moderately admixed populations. Furthermore, our results suggest that a reduced SNP assay, using as few as 10–30 loci, retains accurate probability estimates. This is especially important in cases of failed genotyping, or when cost‐saving measures need to be implemented.

## Author Contributions

**Kathleen A. Dogantzis:** conceptualization (supporting), data curation (lead), formal analysis (lead), funding acquisition (supporting), investigation (lead), methodology (lead), validation (lead), visualization (lead), writing – original draft (lead), writing – review and editing (lead). **Harshilkumar Patel:** methodology (supporting), writing – review and editing (supporting). **Stephen Rose:** methodology (supporting), writing – review and editing (supporting). **Ida M. Conflitti:** resources (lead), writing – review and editing (supporting). **Alivia Dey:** resources (equal), writing – review and editing (supporting). **Tanushree Tiwari:** data curation (supporting), methodology (supporting), writing – review and editing (supporting). **Nadine C. Chapman:** resources (equal), writing – review and editing (supporting). **Samir M. Kadri:** resources (equal), writing – review and editing (supporting). **Harland M. Patch:** resources (equal), writing – review and editing (supporting). **Elliud M. Muli:** resources (equal), writing – review and editing (supporting). **Abdulaziz S. Alqarni:** resources (equal), writing – review and editing (supporting). **Michael H. Allsopp:** resources (equal), writing – review and editing (supporting). **Amro Zayed:** conceptualization (lead), funding acquisition (lead), writing – original draft (supporting), writing – review and editing (equal).

## Conflicts of Interest

The authors declare no conflicts of interest.

## Supporting information


**Figure S1.** Diagram outlining the steps of feature selection, assay development and assay validation. First, we preprocessed SNPs to select markers highly differentiated (*F*_*ST*_ > 0.8) between the African lineage and the remaining lineages. Next, a random forest classification model was used to determine the informativeness of SNPs in discriminating *scutellata*‐hybrids from non‐*scutellata*‐hybrids. A grid search method was used to select the parameters of the random forest model, and the model was then trained on 177 samples (Table S1). Feature importance was measured to identify the top markers over 30 replicates. We identified 824 markers as candidates for assay development, of which 249 met the design requirements and 113 were successfully included in the assay. To validate the assay, we genotyped 1263 validation samples collected from North America, South America and Australia, and estimated their probability of *scutellata*‐hybrid classification using a support vector classifier (SVC). In addition to the validation samples, we tested 117 previously genome‐sequenced samples (Tables S1 and S2). The parameters of the SVC were chosen using a grid search method, and the model was trained on 177 samples (Table S1).
**Figure S2.** ADMIXTURE results for native honey bee samples. Patterns of ancestry and admixture for all (*N* = 243) native *Apis mellifera* samples, grouped into ancestral lineages, as estimated with the program AMDIXTURE. Vertical bars represent individual bees and coloured segments represent the proportion of ancestry estimated for *K* = 3–7 genetic clusters.
**Figure S3.** Principal component analysis of honey bee samples. Samples cluster broadly into representative lineages and populations, with admixed samples clustering outside of lineage groups. The *scutellata*‐hybrid samples cluster closely with A‐lineage samples, while North American commercial colony samples cluster closely with C‐lineage samples.
**Figure S4.** Metrics of model performance of the linear SVC classifier. (A) A confusion matrix, which shows the predicted classification of samples by the trained model relative to their known classification. This model was tested on previously genome‐sequenced honey bees (*n* = 117) (*n* = 88 testing samples and *n* = 29 reference *scutellata*‐hybrids). Samples classified as *scutellata*‐hybrid with > 20% probability were labelled *scutellata*‐hybrid, and samples below this threshold were labelled non‐*scutellata*‐hybrid. All samples were correctly classified at the 20% threshold. (B) The receiver operating characteristic (ROC) curve, which illustrates the performance classification of the model at all classification thresholds. The dashed blue line is the performance of a random model, while the solid green line is the ROC curve for the trained model tested on the reference (*N* = 117) samples. (C). A confusion matrix of the 1263 samples was used to validate the model, whose true classification was known or assumed based on collection location (Table S2). Here, we assume that commercial honey bee samples from North America and all samples from Australia are likely of non‐*scutellata*‐hybrid origin, while *scutellata*‐hybrid honey bees from Brazil and feral honey bees from North America are likely of *scutellata*‐hybrid origin. Samples classified as *scutellata*‐hybrid with > 20% probability were labelled *scutellata*‐hybrid, and samples below this threshold were labelled non‐*scutellata*‐hybrid. One non‐*scutellata*‐hybrid sample was misclassified, while three presumed *scutellata*‐hybrid samples were misclassified. The misclassified non‐*scutellata*‐hybrid sample had a probability of 38.4% to *scutellata*‐hybrid classification but contained several imputed data points due to missing genotype calls (removed from this study). The three presumed *scutellata*‐hybrid samples had a precited probability of < 11% to *scutellata*‐hybrid classification. These samples were collected from Texas where beekeeping with European colonies is prevalent, thus these samples are likely representative of European ancestry colonies (non‐*scutellata*‐hybrid origin). (D) The receiver operating characteristic (ROC) curve for the validation samples (*N* = 1263). The dashed blue line is the performance of a random model, while the solid green line is the ROC curve of the model.
**Figure S5.** Comparison between sample classifications made with a support vector classifier (SVC) and the software ADMXTURE. Both models were trained with 80 informative SNPs using 177 training samples. The SVC model predicts a probability between 0 and 1 and indicates the likelihood of a *scutellata*‐hybrid or non‐*scutellata*‐hybrid classification. The ADMIXTURE model calculates ancestry proportions (*Q* value) to a *scutellata*‐hybrid or non‐*scutellata*‐hybrid cluster. Results depict a boxplot of the probabilities (SVC), or *Q* values (ADMIXTURE), estimated for non‐*scutellata*‐hybrid (A) and *scutellata*‐hybrid (B) samples (Table S2). SH, *scutellata*‐hybrid.

**Table S1.** List of new and previously sequenced reference samples used in this study.
**Table S2.** List of testing and validation samples used to validate the SNP assay and SVC model. This table also indicates which samples were used in the imputation and SNP reduction simulations.
**Table S3.** Information about the 113 markers chosen for the assay.

## Data Availability

All data needed to evaluate the conclusions in the paper are present in the paper and/or the Supporting Information Material. All new honey bee genomes have been deposited on NCBI's Short Read Archive, BioProject: PRJNA1094935. Custom code used for data analysis is publicly available at https://doi.org/10.6084/m9.figshare.25813843.
