# Supplementary material for: Accurate Detection of scutellata‐Hybrids (Africanized Bees) Using a SNP‐Based Diagnostic Assay
Source: Ecol Evol. 2024 Nov 17;14(11):e70554. doi: 10.1002/ece3.70554 (PMC11569865; doi:10.1002/ece3.70554)
Supplement: Supplementary file 1 — Figure S1. Diagram outlining the steps of feature selection, assay development and assay validation. First, we preprocessed SNPs to select markers highly differentiated (F ST > 0.8) between the African lineage and the remaining lineages. Next, a random forest classification model was used to determine the informativeness of SNPs in discriminating scutellata‐hybrids from non‐scutellata‐hybrids. A grid search method was used to select the parameters of the random forest model, and the model was then trained on 177 samples (Table S1). Feature importance was measured to identify the top markers over 30 replicates. We identified 824 markers as candidates for assay development, of which 249 met the design requirements and 113 were successfully included in the assay. To validate the assay, we genotyped 1263 validation samples collected from North America, South America and Australia, and estimated their probability of scutellata‐hybrid classification using a support vector classifier (SVC). In addition to the validation samples, we tested 117 previously genome‐sequenced samples (Tables S1 and S2). The parameters of the SVC were chosen using a grid search method, and the model was trained on 177 samples (Table S1). Figure S2. ADMIXTURE results for native honey bee samples. Patterns of ancestry and admixture for all (N = 243) native Apis mellifera samples, grouped into ancestral lineages, as estimated with the program AMDIXTURE. Vertical bars represent individual bees and coloured segments represent the proportion of ancestry estimated for K = 3–7 genetic clusters. Figure S3. Principal component analysis of honey bee samples. Samples cluster broadly into representative lineages and populations, with admixed samples clustering outside of lineage groups. The scutellata‐hybrid samples cluster closely with A‐lineage samples, while North American commercial colony samples cluster closely with C‐lineage samples. Figure S4. Metrics of model performance of the linear SVC classifier. [file ECE3-14-e70554-s001.docx]

**Supplemental Information for:**

**Accurate detection of *scutellata*-hybrids (Africanized bees) using a SNP-based diagnostic assay.**

Kathleen A. Dogantzis, Harshilkumar Patel, Stephen Rose, Ida M. Conflitti, Alivia Dey, Tanushree Tiwari, Nadine C. Chapman, Samir M. Kadri, Harland M. Patch, Elliud M. Muli, Abdulaziz S. Alqarni, Michael H. Allsopp, Amro Zayed.

Figure S1: Diagram outlining the steps of feature selection, assay development, and assay validation. First, we preprocessed SNPs to select markers highly differentiated (F_ST_ > 0.8) between the African lineage and the remaining lineages. Next, a random forest classification model was used to determine the informativeness of SNPs in discriminating *scutellata*-hybrids from non-*scutellata*-hybrids. A grid search method was used to select the parameters of the random forest model, and the model was then trained on 177 samples (Table S1). Feature importance was measured to identify the top markers over 30 replicates. We identified 824 markers as candidates for assay development, of which 249 met the design requirements, and 113 were successfully included in the assay. To validate the assay, we genotyped 1,263 validation samples collected from North America, South America, and Australia, and estimated their probability of *scutellata*-hybrid classification using a support vector classifier (SVC). In addition to the validation samples, we tested 117 previously genome-sequenced samples (Table S1-2). The parameters of the SVC were chosen using a grid search method, and the model was trained on 177 samples (Table S1).

Figure S2: ADMIXTURE results for native honey bee samples. Patterns of ancestry and admixture for all (N=243) native *Apis mellifera* samples, grouped into ancestral lineages, as estimated with the program AMDIXTURE. Vertical bars represent individual bees and coloured segments represent the proportion of ancestry estimated for K=3-7 genetic clusters.

Figure S3: Principal component analysis of honey bee samples. Samples cluster broadly into representative lineages and populations, with admixed samples clustering outside of lineage groups. The *scutellata*-hybrid samples cluster closely with A-lineage samples, while North American commercial colony samples cluster closely with C-lineage samples.

Figure S4: Metrics of model performance of the linear SVC classifier. A) A confusion matrix, which shows the predicted classification of samples by the trained model relative to their known classification. This model was tested on previously genome-sequenced honey bees (n=117) (n=88 testing samples and n=29 reference *scutellata*-hybrids). Samples classified as *scutellata*-hybrid with >20% probability were labelled *scutellata*-hybrid, and samples below this threshold were labelled non-*scutellata*-hybrid. All samples were correctly classified at the 20% threshold. B) The receiver operating characteristic (ROC) curve, which illustrates the performance classification of the model at all classification thresholds. The dashed blue line is the performance of a random model, while the solid green line is the ROC curve for the trained model tested on the reference (N=117) samples. C). A confusion matrix of the 1,263 samples used to validate the model, whose true classification was known or assumed based on collection location (Table S2). Here, we assume that commercial honey bee samples from North America and all samples from Australia are likely of non-*scutellata*-hybrid origin, while *scutellata*-hybrid honey bees from Brazil and feral honey bees from North America are likely of *scutellata*-hybrid origin. Samples classified as *scutellata*-hybrid with >20% probability were labelled *scutellata*-hybrid, and samples below this threshold were labelled non-*scutellata*-hybrid. One non-*scutellata*-hybrid sample was misclassified, while three presumed *scutellata*-hybrid samples were misclassified. The misclassified non-*scutellata*-hybrid sample had a probability of 38.4% to *scutellata*-hybrid classification but contained several imputed data points due to missing genotype calls (removed from this study). The three presumed *scutellata*-hybrid samples had a precited probability of <11% to *scutellata*-hybrid classification. These samples were collected from Texas where beekeeping with European colonies is prevalent, thus these samples are likely representative of European ancestry colonies (non-*scutellata*-hybrid origin). D) The receiver operating characteristic (ROC) curve for the validation samples (N=1263). The dashed blue line is the performance of a random model, while the solid green line is the ROC curve of the model.

Figure S5: Comparison between sample classifications made with a support vector classifier (SVC) and the software ADMXTURE. Both models were trained with 80 informative SNPs using 177 training samples. The SVC model predicts a probability between zero to one and indicates the likelihood of a *scutellata*-hybrid or non-*scutellata*-hybrid classification. The ADMIXTURE model calculates ancestry proportions (Q value) to a *scutellata*-hybrid or non-*scutellata*-hybrid cluster. Results depict a boxplot of the probabilities (SVC), or Q values (ADMIXTURE), estimated for non-*scutellata*-hybrid (A) and *scutellata*-hybrid (B) samples (Table S2). SH, *scutellata*-hybrid.

Table S1. (Separate file)

List of new and previously sequenced reference samples used in this study.

Table S2. (Separate file)

List of testing and validation samples used to validate the SNP assay and SVC model. This table also indicates which samples were used in the imputation and SNP reduction simulations.

Table S3. (Separate file)

Information about the 113 markers chosen for the assay.
